# Characterization of biologically active exopolysaccharide produced by *Streptomyces* sp*.* NRCG4 and its anti-Alzheimer efficacy: in-vitro targets

**DOI:** 10.1186/s43141-023-00530-9

**Published:** 2023-07-04

**Authors:** Manal G. Mahmoud, Mohamed E. El Awady, Manal S. Selim, Abeer Y. Ibrahim, Faten M. Ibrahim, Sahar S. Mohamed

**Affiliations:** 1 Microbial Biotechnology Department, National Research Centre, 33 Bohouth St, Dokki, Giza, 12622 Egypt; 2 Medicinal and Aromatic Plants Research Department, Pharmaceutical and Drug Industries Research Institute, National Research Centre, 33 Bohouth St, Dokki, Giza, 12622 Egypt

**Keywords:** Exopolysaccharide, *Streptomyces*, Anti-Alzheimer, Anti-tyrosinase, Anti-inflammatory, Antioxidant

## Abstract

**Background:**

Exopolysaccharides are extremely powerful molecules with a wide range of uses in pharmaceuticals due to their structural and compositional complexity. Marine microorganisms often produce bioactive substances with novel functions and structures because of their special living conditions. Polysaccharides from marine microorganisms are of interest to new drug discovery.

**Results:**

The current research focused on the isolation of bacteria from Red Sea, Egypt, that have the ability to produce a new natural exopolysaccharide in order to be examined in treating Alzheimer’s illness to obviate side effects of synthetic drugs. Properties of exopolysaccharide (EPS) produced by an isolated *Streptomyces* strain were investigated for its capability to play as anti-Alzheimer. This strain was identified morphologically, physiologically, and biochemically and actually was confirmed by molecularly 16S rRNA analysis as *Streptomyces* sp. NRCG4 with accession number MK850242. The produced EPS was fractionated by precipitation 1:4 volumes of chilled ethanol and the third major fraction (1:3) listed as NRCG4, and then the functional groups, MW, and chemical evaluation have been detected via Fourier-transform infrared (FTIR), high-performance gel permeation chromatography (HPGPC), and high-performance liquid chromatography (HPLC). The findings showed that NRCG4 was an acidic EPS composed of mannuronic acid, glucose, mannose, and rhamnose in a molar ratio of 1.2:1.5:2.8:1.0, respectively. NRCG4 Mw was determined to be 4.25 × 10^5^ gmol^−1^ and the Mn to be 1.97 × 10^5^ gmol^−1^. Also, the NRCG4 included uronic acid (16.0%) and sulfate (0.0%), but no protein was found. In addition, antioxidant and anti-inflammation activity was measured through various methods. This study confirmed that NRCG4 exopolysaccharide exerted anti-Alzheimer’s characters via inhibition of cholinesterase and tyrosinase as well as anti-inflammatory and antioxidant abilities. Additionally, it occurred a potential role in the suppression of Alzheimer’s disease risk factors through its antioxidant (metal chelation, radical scavenging capability), anti-tyrosinase and anti-inflammatory characteristics. The anti-Alzheimer’s disease efficacy of NRCG4 exopolysaccharide may be assigned to its unique determined chemical composition.

**Conclusions:**

The present study highlighted those exopolysaccharides could be harnessed to improve pharmaceutical industry (anti-Alzheimer, anti-tyrosinase, anti-inflammatory, and antioxidant agents).

## Background

Natural products and their derivatives account for more than half of all medications used in modern medicine. Conventional drug research and development is highly expensive and complex due to the low success rate and large capital investment requirements. Exopolysaccharides (EPSs) produced from microorganisms can contribute in treating various diseases. In recent decades, major attention has been focused on naturally produced polysaccharides, due to changes in monosaccharide composition and sequence, type of linkage, polymerization, and branching degree. Microbial EPSs displayed a wide range of physicochemical and rheological properties. This range of features drew attention to the industrial potential of EPSs in various fields [1, 2]. They were documented for various activities; they were reported as antioxidant agent, cyclooxygenases inhibitor that recommend their role as anti-inflammatory agent [3], acetylcholinesterase inhibitors [4], and anti-Alzheimer’s agent [4, 5]. Approximately 75% and 60% in respect to 70% of known compounds had been produced by actinomycetes that are employed in agriculture and medicine, respectively [6, 7]. Streptomycetes’ heredity, growth, and metabolic regulation have all become research centers in recent years. As a result, streptomycetes are the most commonly observed bacteria in the fermentation of active pharmaceutical drugs [8, 9]. Alzheimer’s disease (AD) is a terrible neurodegenerative disorder characterized by memory and cognition loss, difficulty completing daily activities, and a variety of behavioral and neuropsychiatric diseases [10]. AD is the most frequent form of dementia in the elderly. Every 5 years of age, the percentage of people with Alzheimer’s disease increases by a factor of two. As a result, the disease affects 1% of 60-year-olds and 30% of 85-year-olds. Cell loss, abundant neurofibrillary tangles, dystrophic neuritis, amyloid precursor protein, amyloid deposits, increased activation of pro-death genes and signaling pathways, impaired energy metabolism, mitochondrial dysfunction, chronic oxidative stress, and DNA damage are all hallmarks of Alzheimer's [11]. This work was designed to evaluate the capacities of naturally produced exo-polysaccharide in facing Alzheimer’s disease risk factors with characterization of the produced polysaccharide and identification of its microbial source.

## Methods

### Chemicals

1,1-Diphenyl-2-picryl-hydrazyl (DPPH), polyoxyethylene sorbitan monolaurate (Tween-20), ascorbic acid (vitamin C), butylated hydroxytoluene (BHT), peroxidase, H_2_O_2_, ABTS (2, 2-azino-bis (3-ethylbenz-thiazoline-6-sulfonic acid)), nicotinamide adenine dinucleotide (NADH), nitroblue tetrazolium (NBT), phenazine methosulphate (PMS), sodium nitroprusside (SNP), sulfanilamide, ortho-H_3_PO_4_, naphthylethylene diamine dihydrochloride, diammonium salt, 3-(2-pyridyl)-5,6-bis (4-phenyl-sulfonic acid)-1,2,4-triazine (ferrozine), ferrous chloride, trichloroacetic acid (TCA), potassium ferricyanide, Leuco-2,7-dichlorofluorescien diacetate, hematin, arachidonic acid, cyclooxygenases enzymes (COX-1 from sheep, EC. 1.14.99.1 or human COX-2), acetylcholinesterase enzyme (EC No. 3.1.1.7) type VI-S (from electric eel), acetylthiocholine iodide, 5,5-dithiobis(2-nitrobenzoic acid) (DTNB), butyryl thio-choline iodide, human butyrylcholinesterase enzyme, kojic acid, tyrosinase enzyme, and L-Dopa were purchased from Sigma-Aldrich, USA. Ammonium thiocyanate was purchased from E. Merck. All chemicals and solvents are of analytical grade.

### Sampling and isolation of streptomycetes

Sample was collected from the sediment of the shore (1 cm depth) of the Red Sea, Hurghada (El-Giftoun Island), Egypt. Sample was serially diluted according to Hayakawa and Nonomura [12] and plated on starch nitrate agar medium containing (g/l): starch 10, K_2_HPO_4_ 1.0, MgSO_4_.7H_2_O 0.5, NaCl 0.5, KNO_3_ 2.0, CaCO_3_ 2.0, FeSO_4_.7H_2_O 0.01, agar 20.0 which were dissolved in 750 ml sea water then completed to 1L with 250 ml distilled water, pH 7 [13]. Colonies’ appearance on agar plates was selected for exo-polysaccharide (EPS) production [14]. Further, the actinomycetes isolates were streaked onto starch nitrate agar medium and incubated at 30 °C for 48 h. The *Streptomyces* isolates were selected and picked up based on the morphological features of colonies.

### Production, extraction, and purification of EPS

A loop was full of the strain G4 was inoculated into a 250-ml conical flask containing 50 ml of broth medium containing (g/l) glucose 10.0, tryptone 5.0, yeast extract 5.0, K_2_HPO_4_ 3.0, NaCl 3.0, KH_2_PO_4_ 1.0, MgSO_4_.7H_2_O 0.5, and CaCO_3_ 0.5 which were dissolved in 750-ml sea water then completed to 1L with 250-ml distilled water, pH 7 [15]. This inoculated medium was then incubated on a shaker at 120 rpm for 5 days at 28 °C. The cell-free culture supernatant was obtained by centrifugation at 3466 × *g* for 20; the supernatant was mixed with trichloroacetic acid (TCA) (10%) and keeping it overnight at 4 °C under static conditions, left overnight at 4 °C. Then, centrifuged at 3466 × *g* for 20 min to remove protein and the pH of supernatant was neutralized to 7 with NaOH solution [16]. The EPS-containing solution was completed to 4 volumes with cold absolute ethanol and kept at 4 °C overnight for allowing precipitation of EPS. The precipitated EPS were separated by centrifugation at 3466 × g for 20 min and re-suspended in deionized water then washed twice, and re-precipitated by 4 volume of cold absolute alcohol and dried at 50 °C. For fractionation and major fraction determination, absolute cold ethanol was added 1, 2, 3, and 4 volumes gradually and the precipitated EPS was collected up to date. The major fraction which obtained by three volume was dialyzed against distilled water for 72 h, washed twice with acetone, dehydrated by ether, dried at 40 °C, and named as NRCG4 [17]. The NRCG4 fraction powder was used for structural characterization studies and biological activities.

### Identification of EPS-producing bacterium

The potential EPS-producing isolate G4 was selected based on promising EPS secretion on plate and identified by their morphological, physiological, and biochemical features [18–22] and was confirmed by 16S ribosomal RNA gene technique (16S rRNA). Genomic DNA was extracted from log-phase grown strain G4 by using the modified method of Sharma and Singh [23]. The bacterial genome was isolated and 16 s rRNA gene was amplified using polymerase chain reaction technique using the following primers F-(5′-GAGTTTGATCCTGGCTCAG-3′) and R-(5′-GGTTACCTTGTTACGACTT-3′) based on Gardes and Bruns [24] method. Sequencing was performed by using Big Dye terminator cycle sequencing kit (Applied BioSystems, USA). Sequencing products were resolved on an Applied Bio-systems model 3730XL automated DNA sequencing system (Applied BioSystems, USA). The resulting 16 s rRNA sequence was submitted to GenBank database and compared with the other 16S rRNA sequences in the National Center for Biotechnology Information (https://www.ncbi.nlm.nih.gov/) using the BLAST program. The sequence of other bacterial strains with the greatest similarity to the 16 s rRNA gene of our isolate was selected and aligned for making the suitable phylogenetic tree. The 16 s rRNA gene sequence of our isolate was deposited in the DDBJ/EMBL/GenBank nucleotide sequence databases.

### Chemical analysis of NRCG4

NRCG4 was subjected to complete acid hydrolysis with formic acid (88%) in a sealed tube at 100 °C for 5 h. The sulfate content was estimated by turbidity method using Barium Chloride-Tween 80 reagent according to Dodgson and Price [25]. Uronic acid content was spectrophotometrically analyzed at 525 nm (2401PC 750 Lambda Double Beam UV–Vis; Spectrophotometer Shimadzu, Kyoto, Japan) following the *m-*hydroxybiphenyl colorimetric method [26]. High-performance liquid chromatography (HPLC, Agilate Pack, serics1, 200), equipped with Aminex carbohydrate HP-87C column (300 × 7.8 mm), was applied for monosaccharide composition and calculation of molar ratio using deionized water as a mobile phase at flow rate 0.5 ml/ min [27]. The mass average molar weight (*Mw*) of NRCG4 was determined by high-performance gel permeation chromatography (HPGPC, Agilent 1100 Series System, Hewlett-Packard, Germany) with refractive index (*RI*) detection [28]. The number average molar mass (*Mn*) was determined and polydispersity index (*PI*) was calculated from (*Mw/Mn*) ratio. Therefore, dried NRCG4 (2 mg) was mixed with 200 mg KBr powder, then pressed to form a 1-mm pellet for FTIR spectrum measurement in the range between 400 and 4000 cm^−1^ using FTIR-UNIT Bruker Vector 22 Spectrophotometer [29].

### Acetyl cholinesterase inhibition efficacy assay

NRCG4 were subjected to determine acetyl cholinesterase inhibition efficacy. The enzymatic activity was measured using an adaptation of the method described in Ingkaninan et al. [30]. Five hundred microliters of DTNB (3 mM), 100μL of AChI (15 mM), 275 μL of Tris–HCl buffer (50 mM, pH 8), and 100μL of NRCG4 at 25, 50, 100, 200, and 400 μg ml^−1^ were added to a 1 ml and was used as blank. In the reaction, 25 μL of buffer was replaced by the same volume of an enzyme solution containing 0.28 Uml^−1^. The reaction was monitored for 5 min at 405 nm. Velocities of reaction were calculated. Enzyme activity was calculated as a percentage of the velocities compared to that of the assay using buffer instead of tested inhibitor NRCG4. Inhibitory activity was calculated from 100 subtracted by the percentage of enzyme activity. Data presented here are the average of three replicates. Eserine hemi sulfate salt was used as positive control and it was tested at different concentrations different from samples. The tested eserine concentrations were 0.01, 0.02, 0.04, and 0.08 μg ml^−1^ [31].

### Butyrylcholinesterase inhibitory assessment

Enzymatic activity was analyzed using an adaptation of the method of Ingkaninan Orhan et al. [30]. In sum, 500 μL of the DTNB (3 mM), 100 μl of butyryl thiocholine iodide (15 mM), 275 μl of Tris–HCl buffer (50 mM, pH 8), and 100 μl NRCG4 at different concentrations (25, 50, 100, 200 and 400 μg/ ml) was dissolved in ethyl alcohol (HPLC grade) and was added to a 1 ml cuvette then was used as a blank. In the reaction cuvette, 25 μl of Tris–HCl buffer was replaced by the same volume of butyrylcholinesterase enzyme solution containing 0.28 U ml^−1^. The color developed was monitored for 5 min at 405 nm. The percent of inhibition was calculated.

 

$$\mathrm{Controlabsorbance}-\mathrm{sampleabsorbance})/\mathrm{controlabsorbance}\times100.$$

The IC_50_ was calculated by log-probit analysis. Eserine served as a positive control [32].

### Anti-tyrosinase activity assessment

Tyrosinase inhibition assay (EC1.14.18.1) was performed according to the methods of Liu et al. [33] with slight modifications. Briefly, NRCG4 sample and kojic acid (Sigma, St. Louis, MO, USA) were dissolved in DMSO prepared as 0.1 mg/ ml. The reaction was carried out and (Jasco, serial No. C317961148, Japan) spectrophotometer was used to measure the absorbance at 475 nm. NRCG4 (40 μl each) was dissolved in methanol with 80 μl of phosphate buffer (pH 6.8), 40 μl of tyrosinase enzyme, and 40 μl of L-Dopa (Sigma, St. Louis, MO, USA) which were placed in each well. Each sample was accompanied by a blank that had all the components except for L-Dopa. Kojic acid was used as the standard inhibitor. The percentage of tyrosinase inhibition was calculated as follows:

$$\lbrack(\mathrm{Acontrol}-\mathrm{Asample})/\mathrm{Acontrol}\rbrack\times100$$

where *A*_control_ is the absorbance of the control reaction and *A*_sample_ is the absorbance of the extracts or standard, analyses were run in triplicate.

### Anti-inflammatory activity

The cyclooxygenase inhibition efficacy NRCG4 was performed according to a modified method of Larsen et al. [34] and celecoxib was used as the standard compound. The concentrations of tested materials were 5, 10, 20, 40, 80, and 100 μg/ml whereas they were in ng/ ml for standard, celecoxib, in examination against COX-2. On the other hand, the concentrations of tested materials and standard were 25, 50, 100, 200, and 400 μg/ml against COX-1. Leuco-2, 7-dichlorofluoresce in diacetate (5 mg) was hydrolyzed at room temperature in 1 M NaOH (50 μl) for 10 min, then 1 M HCl (30 μl) was added to neutralize excess NaOH before the resulting 1-DCF was diluted in 0.1 M Tris-buffer, pH 8. Cyclooxygenase enzyme (COX-1 or COX-2) was diluted in 0.1 M Tris-buffer, pH 8, so that a known aliquot gave an absorbance change of 0.05/min in the test reaction. Test samples (or the equivalent volume of methanol, 20 μl) were pre-incubated with enzyme at room temperature for 5 min in the presence of hematin. Premixed phenol, 1-DCF, and arachidonic acid were added to the enzyme mixture to begin the reaction, and to give a final reaction mixture of arachidonic acid (50 μM), phenol (500 μM), 1-DCF (20 μM), and hematin (1 μM) in 1-ml final volume of 0.1 M Tris-buffer, pH 8. The reaction was recorded spectrophotometrically over 1 min at 502 nm. A blank reaction mixture was analyzed in the spectrophotometer reference cell against each test reaction to account for any non-enzymatic activity attributed to the test sample. This blank consisted of the reaction mixture without the addition of enzyme.

### Antioxidant activities

#### Free radical scavenging characters

##### DPPH radical scavenging activity

According to the method of Ibrahim et al. [35] the free radical scavenging activity of NRCG4 using 1,1-diphenyl-2-picryl-hydrazil (DPPH·). DPPH· (0.1 mM) was prepared in methanol. Successive concentration from polysaccharide and standard materials; BHT and Ascorbic Acid were prepared as 25, 50, 100, 200, and 400 μg/ ml in methanol. One milliliter of DPPH· solution was added to 3 ml of each concentration, and then the mixture was shaken vigorously and was allowed to stand at room temperature for 30 min. Control was assayed with the same procedure, except methanol was instead sample. The absorbance was read at 517 nm in a spectrophotometer (Jasco, serial No. C317961148, Japan). According to the following equation, DPPH radical scavenging activity was calculated:

$$\mathrm{DPPH}\cdot\mathrm{scavengingeffect}(\%)=\lbrack(\mathrm A0-\mathrm A1)/\mathrm A0)\times100\rbrack.$$

where *A*_0_ was the absorbance of the control and *A*_1_ was the absorbance in the presence of the sample of polysaccharide.

### ABTS radical cation scavenging activity

Based on the method described by Miller and Rice-Evans [36] and modified by Arnao et al. [37], ABTS radical cation scavenging activity of NRCG4 polysaccharide at different concentrations was estimated and was compared with two standard materials: BHT and ascorbic acid at the same concentrations. Reaction was prepared by adding 0.2 ml of peroxidase (4.4units/ ml), 0.2 ml of H_2_O_2_ (50 μM), and 0.2 ml of ABTS (100 μM) and 1-ml methanol mixed and kept in the dark for 1 h to form a bluish green complex. After dark incubation period, 1 ml of polysaccharide, BHT, and ascorbic Acid at different concentrations were added. Control was prepared by the same procedure without samples. The absorbance at 734 nm was measured to represent the ABTS radical cation scavenging activity and then was calculated as follows:

$$\mathrm{ABTS}\;\mathrm{radical}\;\mathrm{cation}\;\mathrm{scavenging}\;\mathrm{activity}\;(\%)=\lbrack1-(\mathrm{Asample}/\mathrm{Acontrol})\rbrack\times100.$$

### Lipid peroxidation in ammonium thiocyanate medium

The ability of NRCG4 to inhibit lipid peroxidation was determined according to the method of Gulcin et al. [38] with some modifications and was compared with BHT and ascorbic acid to evaluate it. A pre-emulsion was prepared by mixing 175 μg Tween 20, 155 μl linoleic acid, and 0.04 M potassium phosphate buffer (pH 7.0). One milliliter of sample in 99.5% ethanol was mixed with 4.1 ml linoleic emulsion, 0.02 M phosphate buffer (pH 7.8), and distilled water (pH 7.9). The mixed solutions of samples (21 ml) were incubated in screw cap tubes under dark conditions at 40 °C at certain time intervals. 0.1 ml of this mixture was pipetted and added with 9.7 ml of 75% and 0.1 ml of 30% ammonium thiocyanate sequentially. After 3 min, 0.1 ml of 0.02 M ferrous chloride in 3.5% HCl was added to the reaction mixture. The peroxide level was determined by reading daily of the absorbance at 500 nm in a spectrophotometer. The inhibition of lipid peroxidation in percentage was calculated by the following equation:

$$\mathrm{Lipid}\;\mathrm{peroxidation}\;\mathrm{inhibition}\;(\%)=\lbrack1-(\mathrm A0)/\mathrm A1)\rbrack\times100$$

where *A*_0_ was the absorbance of the control reaction and *A*_1_ was the absorbance in the presence of polysaccharide or standard compounds.

#### ***Reduction of ferric ions (Fe***^***3***+^***) power***

The Fe^3+^ reducing power of NRCG4 was assayed according to the method of Oyaizu [39] and was compared with BHT and ascorbic acid as standard materials. Each concentration from tested compounds was mixed with phosphate buffer (2.5 ml, 0.2 M, pH 6.6) and potassium ferricyanide [K_3_Fe (CN) _6_] (2.5 ml, 1%). The mixture was incubated at 50 °C for 20 min. 2.5 ml of TCA (10%) was added to the mixture, which was then centrifuged for 10 min at 1000 × *g* (MSE Mistral 2000, UK, and Serial No.: S693/02/444). The upper layer of solution (2.5 ml) was mixed with methanol (2.5 ml) and FeCl_3_ (0.5 ml, 0.1%), and the absorbance was measured at 700 nm in a spectrophotometer.

#### ***Ferrous ions (Fe***^***2***+^***) chelating capacity***

The Fe^2+^ chelating activity of NRCG4 was estimated according to the method of Dinis et al. [40] and was evaluated by comparing with two standard compounds (BHT and ascorbic acid at the same conditions). Each concentration was added to 2 mM FeCl_2_ (0.05 ml). The reaction was initiated by the addition of 5 mM ferrozine (0.2 ml) and the mixture was shaken vigorously and left standing at room temperature for 10 min. The control contained FeCl_2_ and ferrozine justly. The absorbance of the reaction was read at 562 nm in a spectrophotometer. The percentage of inhibition of ferrozine-Fe^2+^ complex formation was given by the formula:

$$\mathrm{Inhibition}\;(\%)=\lbrack(\mathrm A0-\mathrm A1)/\mathrm A0\rbrack\times100.$$

where *A*_0_ was the absorbance of the control and *A*_1_ was the absorbance in the presence of the sample of polysaccharide and standards.

### Reactive oxygen species (ROS) scavenging capacity

#### Superoxide anion scavenging activity

Measurement of superoxide anion (O^2**−**^) scavenging activity of NRCG4 was based on the method described by Liu et al. [41] with slight modifications [38]. O^2−^ were generated in 3 ml of Tris–HCl buffer (16 mM, pH 8.0) containing 1 ml of NBT (50 μM) solution, 1 ml NADH (78 μM) solution and 1 ml from NRCG4 or standard materials solution at different concentrations were mixed. The reaction was started by adding 1 ml of PMS solution (10 μM) to the mixture. The reaction mixture was incubated at 25 °C for 5 min and was read at 560 nm in a spectrophotometer. Control was prepared with the same procedure without sample. O^2−^ scavenging was calculated using the following formula:

$$\mathrm{TheO}2-\mathrm{scavenging}\%=\lbrack(\mathrm A0-\mathrm A1)/\mathrm A0\rbrack\times100.$$

where *A*_0_ was the absorbance of the control and *A*_1_ was the absorbance of polysaccharide or standard samples.

#### Nitric oxide radical scavenging activity

Using sodium nitroprusside (SNP), the NO· radical scavenging activity of tested material was determined and was compared with two standard materials: BHT and Ascorbic Acid. NO· was generated from SNP in an aqueous solution at physiological pH to produce nitrite ions which were measured by the Greiss reagent [42]. Greiss reagent constitutes 1% sulfanilamide in 5% ortho-H_3_PO_4_ and 0.1% naphthyl ethylene diamine dihydrochloride. The reaction mixture contained from 2 ml of polysaccharide and standard compounds at different concentrations and SNP (10 mM) in phosphate-buffered saline pH 7.4 were incubated at 25 °C for 150 min. After incubation, 1-ml samples of reaction mixtures were removed and were diluted with 1 ml Greiss reagent. The absorbance of these solutions was measured at 540 nm against the corresponding blank solution.

#### Statistical analysis

The data were presented as mean ± SE. Data obtained were analyzed by ANOVA one-way and post hoc for multiple comparisons using the IBM-SPSS statistics program (version 25) at *P* ≤ 0.05, *t*-test (*n* = 3 replicates) was used in comparisons.

## Results

### Screening of streptomycetes for production of EPSs

Streptomycetes are the most common bacteria used in the fermentation of active medicinal substances. Therefore, totally, ten isolates were from the sediment of the Red Sea, Hurghada (El-Giftoun Island), Egypt. Two isolates (G1 and G4) only could highly produce polysaccharide (NRCG1 and NRCG4) which were 6.54 and 7.76 g/ l, respectively (Table 1).

**Table 1:** Dry weight, EPSs, and productivity of EPSs of different isolates

Samples	Dry weight (g/l)	EPS (g/l)	Productivity (%)
G 1	6.08	3.32	54.6
G 2	7.76	**6.54**	84.2
G 3	4.89	5.03	102.8
G 4	5.8	**7.76**	133.7
G 5	6.4	2.33	36.4
G 6	4.6	5.31	115.4
G 7	4.91	2.29	64.6
G 8	5.09	2.08	40.8
G 9	3.83	2.75	71.8
G 10	6.36	4.23	66.5

### Examination of EPSs as inhibitors for neurotransmitters degradation

Acetyl cholinesterase inhibition efficacy percentage was done for different concentrations of EPSs produced by streptomycetes isolates (G1 and G4). Results showed that EPS from G4 has increasing in acetyl cholinesterase inhibition activity percentage reached to 79.8% at 1000 ppm (Table 2).

**Table 2:** Acetyl cholinesterase inhibition (%) of EPSs preliminary test

Isolates	Acetyl cholinesterase inhibition (%)			
	25 ppm	50 ppm	100 ppm	200 ppm
G1	7.8	11.4	16.2	23.4
G4	22.2	34.2	55.8	79.8

### Identification of the promising streptomyces isolate

**Fig. 1:**
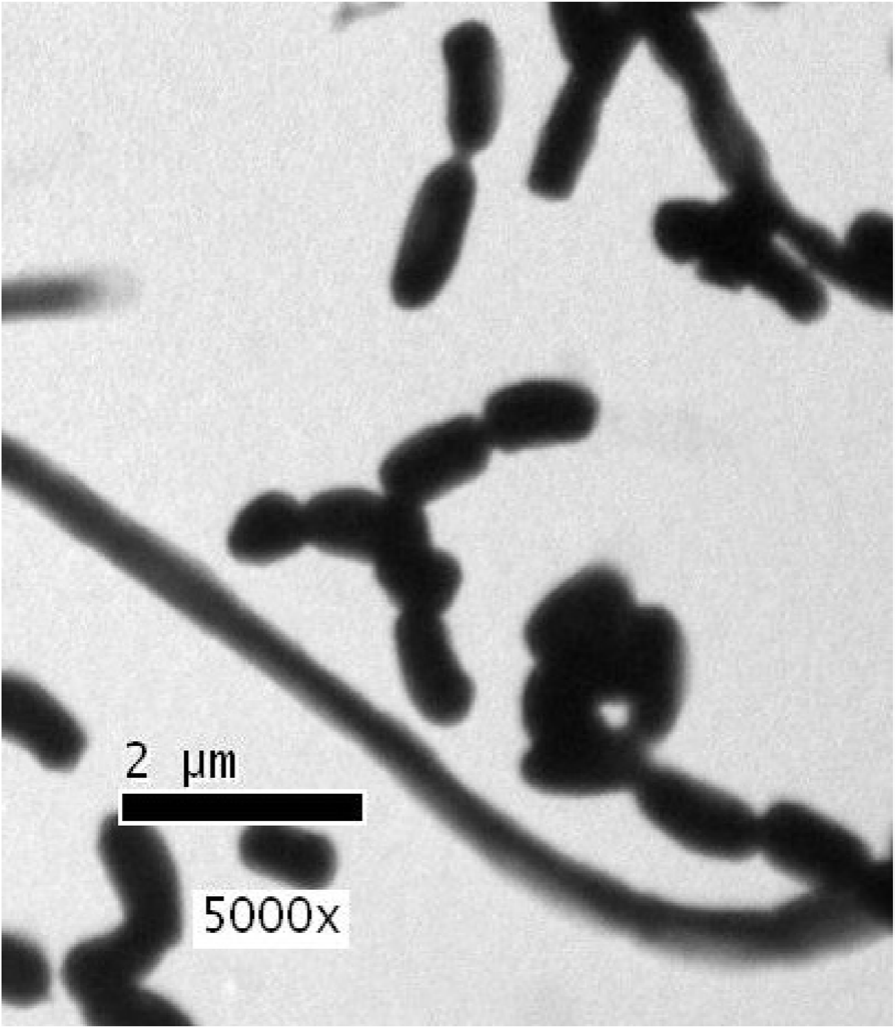
TEM photomicrograph of G4 showing smooth spore surface

**Fig. 2:**
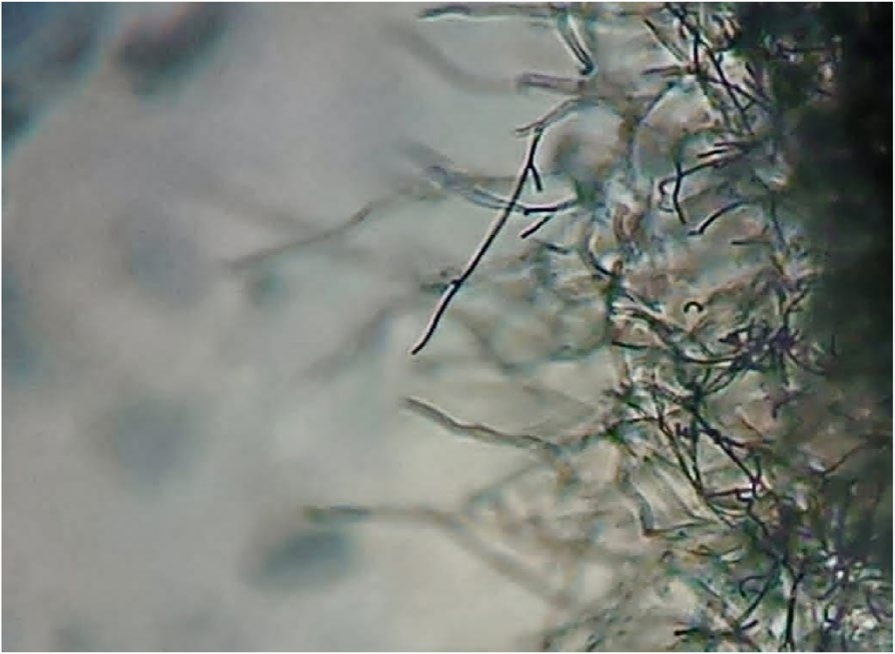
Photomicrograph showing straight/flexuous sporophores hyphae

**Fig. 3:**
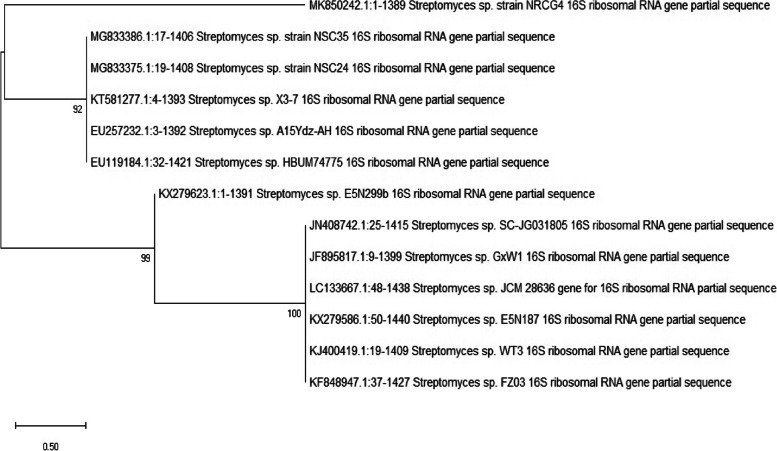
Phylogenetic tree of the partial sequence of 16S rRNA of the local isolate *Streptomyces* sp. NRCG4 respects to closely related sequences available in Gen Bank databases

The most isolate (NRCG4) has high acetyl cholinesterase inhibition activity which was subjected to morphological (Figs. 1 and 2) and physiological and biochemical characteristics (Table 3). Whereas, the comparative analysis of ribonuclease-resistant oligonucleotides of the 16S ribosomal RNA is the most valuable technique for establishing the relatedness of higher taxa (16 s rRNA) [43]. Therefore, phylogenetic analysis based on the 16S rRNA gene sequence of strain NRCG4 was compared to reference 16S rRNA quality arrangement accessible in the GenBank and EMBL database acquired from the National Centre of Biotechnology Data database utilizing BLAST search (http://ncbi.nlm.nih.gov/BLAST/). Therefore, it was determined to be *Streptomyces* sp. NRCG4 with accession number MK850242. Figure 3 shows phylogenetic tree of the partial sequence of 16S rRNA of the local isolate *Streptomyces* sp. NRCG4 respects to closely related sequences found in GenBank databases. The sequences of other streptomyces strains having the highest similarity to the 16 s rRNA gene of our isolate, ranging from 92 to 100 percent, were chosen and linked to create an appropriate phylogenetic tree.

**Table 3:** Different characteristics of streptomycete isolate (G4)

Morphological and cultural characteristics																
Spore chain morphology				Spore surface ornamentation				Color of spore mass		Pigmentation of substrate mycelium				Diffusible pigment		
**Straight/flexuous > 40**				**Smooth**				**Gray**		**grayish white**				**-ve**		
Physiological and biochemical characteristics																
Melanin pigment production					Degradation activities							Nitrate reduction			H_2_S production	
Peptone iron		Tyrosine			Xanthine		Elastin		Arbutin			** + ve**			** + ve**	
**-ve**		**-ve**			**-ve**		**-ve**		** + ve**							
Utilization of sugars																
D-fructose	Sucrose		Rhamnose			D-mannitol		D-xylose	Raffinose		I-inositol		Galactose			L-arabinose
**-ve**	** + ve**		**-ve**			**-ve**		** + ve**	**-ve**		**-ve**		** + ve**			** + ve**

### Isolation, partial purification, and chemical analysis of NRCG4

**Fig. 4:**
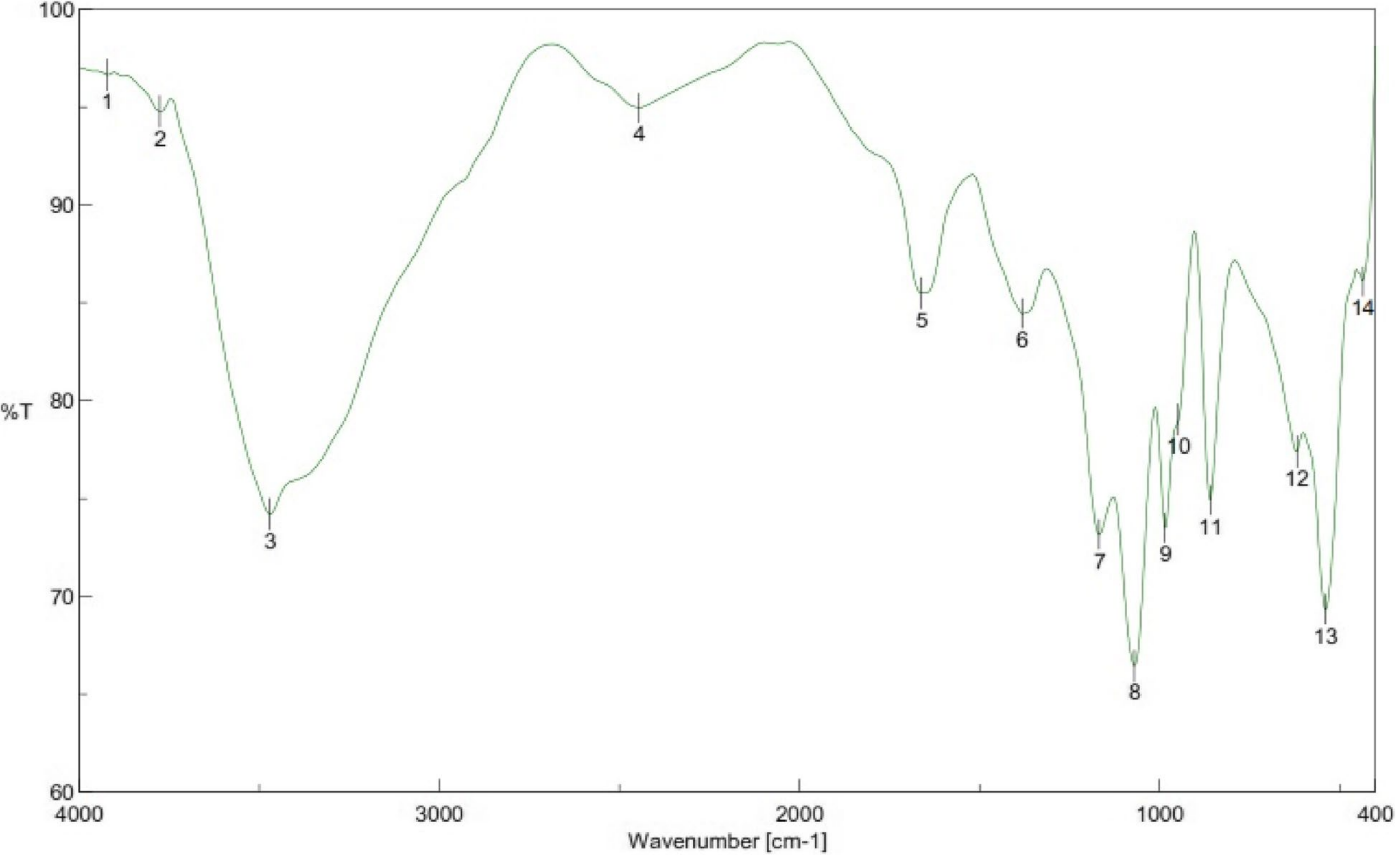
IR spectrum of NRCG4

EPS production was high (7.76 g/l) by *Streptomyces* sp. NRCG4. Ethanol precipitation and dehydration with acetone and diethyl ether were used to extract crude NRCG4 from fermented broth*.* The crude NRCG4 was subjected to another precipitation step with chilled absolute ethanol. The major fraction was obtained by three volumes of absolute ethanol which was dried under vacuum at 40 °C to generate NRCG4, which was employed in further analyses. It showed a negative Bradford test result without absorption in the UV spectrum at 280 nm or 260 nm, confirming the lack of protein and nucleic acid. It also included 16.0% uronic acid and 0.0% sulfate. A significant band at 3469.31 cm^−1^ was seen in the FT-IR spectrum, which was attributed to carbohydrates O–H stretching vibration. C–H stretching vibration caused the 2444.33 cm^−1^ band. The presence of COO^−^ groups was revealed by a symmetrical extended peak about 1380.78 cm^−1^ (Fig. 4). C = O vibrations were blamed for the significant absorption at 1660.41 cm^−1^. Furthermore, distinctive absorptions at 859.32 cm^−1^ suggest that α-configurations were present in NRCG4 at the same time [44, 45].

**Fig. 5:**
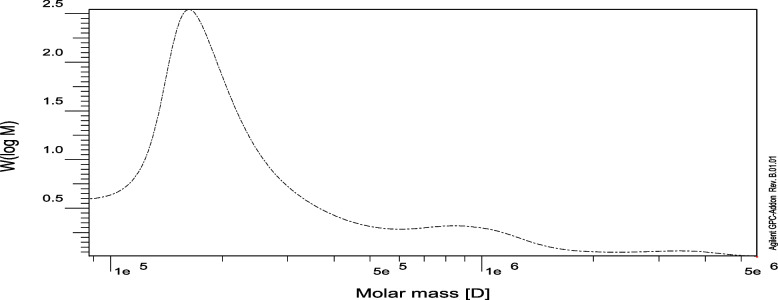
Molecular weight of NRCG4

HPLC was used to examine the chemical structure of NRCG4 and compare it to monosaccharide standards. NRCG4 was made up of 1.2:1.5:2.8:1.0 molar ratios of mannuronic acid, glucose, mannose, and rhamnose. For the sections of peaks that lie within the peak ranges, the molecular weight of NRCG4 was computed. NRCG4 Mw was found to be 4.25 × 10^5^ gmol^−1^ and the Mn was found to be 1.97 × 10^5^ gmol^−1^. The polydispersity index (Mw/Mn) is a metric for the molecular weight distribution’s width 2.1 (Fig. 5). *Streptomyces* sp. is thus considered common host bacteria for industrial and medicinal applications.

### Neurotransmitter’s degradation inhibitory characters of NRCG4

#### Acetyl cholinesterase inhibitory action

Acetylcholinesterase inhibitory property assessment has been used to determine the ability of exopolysaccharide to prevent neurotransmitter degradation that could provoke brain function in optimum. NRCG4 possessed significant and concentration-dependent AChE inhibitory action (Table 4) as its inhibition percentage started with 33.72 ± 1.28% at 25 μg/ ml to 79.98 ± 2.50% at 400 μg/ ml. The calculated inhibitory action for 50% of enzyme activity value of NRCG4 was 87.29 μg/ ml.

**Table 4:** Acetylcholine esterase and butyrylcholinesterase inhibition activity of different concentrations (25–400 μg/ mL) of NRCG4

**Concentrations (µg/ ml)**	NRCG4	
	**Acetylcholine esterase**	**Butyrylcholine esterase**
**25**	33.72 ± 1.28	38.34 ± 0.66
**50**	46.34 ± 1.66	43.69 ± 1.67
**100**	55.81 ± 1.82	60.42 ± 1.58
**200**	63.85 ± 1.15	72.53 ± 1.59
**400**	79.98 ± 2.50	85.26 ± 1.74
**IC**_**50**_	**87.29**	**71.38**
**Eserinhemisulphate IC**_**50**_ was 0.03 µg/ml		

### Butyryl cholinesterase inhibitory action

The inhibitory action of polysaccharide-6 on BChE was evaluated in in vitro assay and is summarized in Table 4. NRCG4 polysaccharide exhibited potential inhibitory action on BChE and the inhibition was enhanced by increasing the concentration of NRCG4. Obtained results indicated that NRCG4 IC_50_ value for butyrylcholinesterase was 71.38 μg/ ml.

### Anti-tyrosinase inhibitory activity

L-tyrosine is used as the substrate in the determination of tyrosinase inhibitory activity of NRCG4. Data are presented in Table 5 as percentage of inhibition at gradual concentrations considering incubation time as well as IC_50_ values in comparison with kojic acid, standard material. The inhibitory action of NRCG4 on tyrosinase was increased gradually with the increased consecutive concentrations and time-dependently. The greatest tested concn. (400 μg/ml) presented a promising tyrosinase inhibitory activity (98.59 ± 1.40%) after 40 min of incubation. In addition, kojic acid which was used as a positive control showed an inhibition level of 100% to tyrosinase at the same time and concentration with insignificant difference with tested polysaccharide. The minimum inhibitory action was produced with the minimum concentration (25 μg/ml) and it was increased time-dependently from 6.45% after 10 min of incubation to 45.13% after 20 min and continued its increasing to reach 71.23% after incubation for 40 min; the same trend of results was also obtained by the other concentrations. The second concentration (50 μg/ml) started the inhibition with 12.15% and elevated to 51.64% by increasing the time to 20 min and was maximized to 80.54% after 40 min of incubation. Accordingly, the suppressor action on tyrosinase was maximized from 21.64% for the third concentration (100 μg/ml) after 10 min to 67.95% after 20 min and elevated to 91.77% after 40 min of incubation. In parallel, tyrosinase was completely inhibited (98.59%) after 40 min of incubation time at 400 μg/ml whereas it started with 25.39% and magnified to 75.12% by duplication of incubation time to 20 min, insignificant differences were observed between kojic acid, reference material, at the two times (20 and 40 min), *P* ≤ *0.05*. The recorded IC_50_ values at the two evaluated times (20 and 40 min) were 47.57 and 17.63 μg/ ml for NRCG4 and 99.62 and 18.07 μg/ml for kojic acid at the two evaluated times 20 and 40 min, respectively.

**Table 5:** Tyrosinase inhibition efficacy of different concentrations (25–400 μg/ mL) of NRCG4 in respect to kojic acid, reference material

Concentrations (μg/ml)	NRCG4			Kojic acid		
	**10 min**	**20 min**	**40 min**	**10 min**	**20 min**	**40 min**
**25**	6.45 ± 0.45^a^	45.13 ± 0.87 ^a^	71.23 ± 1.77	9.34 ± 0.66	37.55 ± 1.45	70.32 ± 1.68
**50**	12.15 ± 0.85^a^	51.64 ± 1.00 ^a^	80.54 ± 1.615	16.48 ± 0.52	43.11 ± 0.95	80.41 ± 1.59
**100**	18.37 ± 0.83^a^	59.11 ± 1.41 ^a^	85.67 ± 2.67	25.88 ± 1.17	52.37 ± 1.63	85.75 ± 2.20
**200**	21.64 ± 0.69^a^	67.95 ± 1.89 ^a^	91.77 ± 2.78	32.64 ± 0.68	61.84 ± 1.16	95.12 ± 1.33
**400**	25.39 ± 0.60^a^	75.12 ± 2.88 ^a^	98.59 ± 1.40	40.35 ± 1.65	70.23 ± 1.77	100.0 ± 0.00
**IC**_**50**_	**765.19**^**a**^	**47.57 **^**a**^	**17.63**	**566.00**	**99.62**	**18.07**

Data presented as mean ± SE. One-way ANOVA was used for data analysis (*n* = 3, *P* < 0.05). Data are followed with small letter a means significant difference with kojic acid at the same time and concentration

### Anti-inflammatory property of NRCG4

Anti-inflammatory efficacy of NRCG4 was evaluated as its inhibitory action on cyclooxygenases enzymes, COX-2 and COX-1, in respect to celecoxib as a reference drug. Celecoxib produced COX-1 inhibition percentage started with 44.12% ± 1.74 at 25 μg/ml and increased continuously to reach 81.61 ± 1.73% for 1000 μg/ ml whereas it was effective in inhibiting COX-2 as it recorded inhibition percentage ranged from 28.53 ± 1.02% at 5 μg/ ml to 81.61 ± 1.67% at 100 μg/ ml. On the contrary, data presented in Table 6 showed the selective inhibitory action of NRCG4 against COX-2. NRCG4 inhibited COX-2 in concentration-dependent manner (10.57 ± 1.00% at 5 μg/ ml till reaching 83.34 ± 1.34% at 100 μg/ml). It was evident from the obtained results that the inhibition of COX-2 by 50% required 28.91 μg/ml of NRCG4 which was greater than celecoxib (IC_50_; 27.45 ng/ ml). In parallel, NRCG4 exhibited a greater safety margin on physiological system as it showed greater IC_50_ value for COX-1 (45.23 μg/ml) than reference drug (IC_50;_ 40.61 μg/ml).

**Table 6:** COX-2 and COX-1 inhibition by different concentrations of NRCG4 and standard drug

COX-2			COX-1		
**Concentrations (µg/ml)**	**Inhibition percentage (%)**		**Concentrations (µg/ ml)**	**Inhibition percentage**	
	**NRCG4**	**Celecoxib**			
				**Polysaccharide**	**Celecoxib**
**5**	10.57 ± 1.00^a^	28.53 ± 1.02	**25**	46.71 ± 0.98	44.12 ± 1.74
**10**	30.37 ± 1.52^a^	33.84 ± 1.31	**50**	57.48 ± 1.52	58.34 ± 1.38
**20**	42.15 ± 1.63^a^	45.21 ± 0.99	**100**	66.71 ± 1.36	65.67 ± 1.06
**40**	68.99 ± 1.85^a^	61.45 ± 2.07	**250**	72.19 ± 1.04	70 ± 1.08
**80**	77.62 ± 2.00^a^	72.11 ± 1.48	**500**	75.44 ± 1.11	76.22 ± 0.96
**100**	83.34 ± 1.34	81.61 ± 1.67	**1000**	80.17 ± 1.42	81.61 ± 1.73
**IC**_**50**_	**28.91**	**27.45**	**IC**_**50**_	**45.23**	**50.61**

Data are presented as mean of three triplicates ± SE. One-way ANOVA was used for data analysis (*n* = 3, *p* < *0.05*). Data are followed with small letter a which means significant difference with celecoxib at the same concentration

### Oxidative stress inhibitory characters

#### DPPH free radical scavenging ability

The ability of NRCG4 to scavenge free radicals was assessed using the DPPH test in comparison to two standard components, ascorbic acid and BHT as shown in Table 7 (a). The presented results showed that NRCG4 appeared promising DPPH radical scavenging ability that was significantly increased when NRCG4 concentration was increased from 25 to 400 μg/ml (46.32 ± 1.68% and 88.26 ± 1.74%, respectively). On the other hand, BHT exhibited 80.46 ± 2.52% scavenging percentage increased to 98.32 ± 1.69% at the same two concentrations (*P* < *0.05*). Considering IC_50_ values, the scavenging ability of NRCG4 and standard materials against DPPH radical occurred in the following order Ascorbic acid > BHT > NRCG4 with significant differences (*P* < *0.05*).

**Table 7:** Scavenging of DPPH, ABTS, SOR and NO ability, reduction capability, Fe ^2+^ ion chelation property, and lipid peroxidation inhibition of NRCG4 as compared with two reference standards ascorbic acid and BHT

Concentration (μg/mL)	NRCG4	Ascorbic acid	BHT
a. **DPPH**			
25	46.32^b^ ± 1.68	75.17 ± 1.30	80.46 ± 2.52
50	79.08^ab^ ± 3	90.31 ± 2.31	96.10 ± 2.90
100	81.46^ab^ ± 2.48	95.28 ± 3.20	97.88 ± 2.11
200	84.88^ab^ ± 2.12	97.50 ± 2.00	98.00 ± 2.00
400	88.26^ab^ ± 1.74	100.00 ± 0.00	98.32 ± 1.69
**IC**_**50**_	**40.12**	**31.34**	**28.18**
**b. ABTS**			
25	47.94 ± 0.56^ab^	66.32 ± 2.68	62.18 ± 1.81
50	61.11 ± 1.56 ^ab^	81.94 ± 1.06	83.31 ± 1.69
100	76.55 ± 1.90 ^ab^	90.89 ± 1.90	90.01 ± 1.50
200	89.69 ± 1.73 ^ab^	95.25 ± 2.25	94.00 ± 1.75
400	98.69 ± 0.51	100.00 ± 0.0	100.00 ± 0.0
**IC**_**50**_	**31.91 **^**ab**^	**18.61**	**23.44**
**c. Reduction capability**			
25	0.166 ± 0.04 ^ab^	0.392 ± 0.004	0.287 ± 0.013
50	0.210 ± 0.03 ^ab^	0.583 ± 0.013	0.492 ± 0.05
100	0.352 ± 0.03 ^ab^	0.64 ± 0.04	0.534 ± 0.01
200	0.450 ± 0.03 ^ab^	0.722 ± 0.05	0.588 ± 0.02
400	0.558 ± 0.02 ^ab^	0.780 ± 0.003	0.603 ± 0.03
**IC**_**50**_	**150.34 **^**ab**^	**85.17**	**88.11**
**d. Fe 2 + ion chelation ability**			
25	35.43 ± 1.57^ab^	62.99 ± 1.01	52.41 ± 1.59
50	39.21 ± 2.00^ab^	82.33 ± 1.67	87.93 ± 2.00
100	43.76 ± 1.78^ab^	91.50 ± 2.52	94.16 ± 2.11
200	46.10 ± 1.90^ab^	94.75 ± 1.95	97.50 ± 2.05
400	47.95 ± 1.96^ab^	98.19 ± 1.82	99.09 ± 0.90
**IC**_**50**_	**416.38**^**ab**^	**16.36**	**18.87**
**e. Lipid peroxidation Inhibition capacity**			
25	39.48 ± 0.52^ab^	64.73 ± 2.20	60.31 ± 1.69
50	43.69 ± 0.79 ^ab^	74.58 ± 3.00	76.41 ± 0.59
100	48.77 ± 1.78 ^ab^	88.74 ± 3.20	87.51 ± 1.60
200	51.37 ± 0.63 ^ab^	94.37 ± 2.75	93.75 ± 1.75
400	53.44 ± 1.78 ^ab^	100.00 ± 0.00	100.00 ± 0.00
**IC**_**50**_	**190.45 **^**ab**^	**18.33**	**19.65**
**f. O**^**2−**^** radicals scavenging capacity**			
25	65.91b ± 2.09	66.21 ± 1.79	73.22 ± 2.78
50	73.13ab ± 1.87	80.37 ± 2.30	83.61 ± 3.36
100	74.73ab ± 3.03	95.34 ± 2.66	97.21 ± 2.79
200	79.02ab ± 1.98	97.25 ± 1.75	97.88 ± 1.55
400	81.27ab ± 2.73	97.75 ± 2.25	98.11 ± 1.89
**IC**_**50**_	**14.22 **^**ab**^	**12.35**	**11.01**
**g. NO scavenging capacity**			
25	17.89 ± 0.90 ^ab^	64.39 ± 1.61	65.99 ± 2.00
50	25.87 ± 0.90 ^ab^	78.68 ± 1.70	77.92 ± 2.00
100	39.20 ± 1.27 ^ab^	86.15 ± 1.95	88.72 ± 2.00
200	45.59 ± 1.13 ^ab^	90.25 ± 2.00	91.75 ± 1.55
400	58.64 ± 1.20 ^ab^	93.27 ± 2.73	95.88 ± 1.89
**IC**_**50**_	**322.96 **^**ab**^	**19.96**	**18.93**

*BHT*, butylated hydroxy toluene. Data are presented as mean ± SE. One-way ANOVA was used for data analysis (*n* = 3, *P* < *0.05*). Data are followed with small letter a means significant difference with ascorbic Acid and b means significant difference with BHT

### ABTS cation radicals scavenging capability

The ABTS/ H_2_O_2_ discoloration method was used to test the ability of NRCG4 and reference materials to scavenge ABTS radicals at different concentrations (Table 7 (b)). NRCG4 displayed powerful scavenging ABTS^+^ cation radical character as compared to two reference materials, as evidenced by a discolored bluish-green complex of ABTS/H_2_O_2_ that increased concentration-dependently. NRCG4 showed high activity at the lowest concentration 25 μg/ml, (47.94 ± 0.56%) which increased gradually to 98.69 ± 0.51% by increasing concentration to 400 μg/ ml, with respect to vitamin C (66.32 ± 2.68% and 100 ± 0.00% for the same concentrations, respectively) and BHT (62.18 ± 1.81% and 100 ± 0.01%, respectively) (*P* < *0.05*). The IC_50_ of NRCG4 in ABTS + system was 31.91 μg/ml, whereas BHT and ascorbic acid exerted 23.44 μg/ml and 18.61 μg/ml, respectively.

### Reduction capability

The Fe^3+^ reductive capability of NRCG4 was assessed using Fe^3+^-Fe^2+^ transformation test and was compared to ascorbic acid and BHT as reference materials. Ferric ions were reduced to ferrous ions in potassium ferricyanide method when more electrons are donated by antioxidant components. Table 7 (c) clears that, as a reducing agent, NRCG4 had a limited effect with significant changes as concentration was changed. Furthermore, reduction ability of NRCG4 was increased significantly with increasing concentrations. NRCG4 exhibited reductive ability (absorbance; 0.558 ± 0.02) and it remained lower than vitamin C (0.780 ± 0.003) and BHT (0.603 ± 0.03). Regarding efficacy arrangement, NRCG4 and standard materials reduction ability were ordered as follows; NRCG4 < BHT < vitamin C (*P* < *0.05*).

### ***Fe ***^***2***+^***ion chelation ability***

For testing NRCG4 and standard materials (vitamin C and BHT) chelating efficacy, ferrous ion (Fe^+^) chelation was evaluated among the transition metals by generating complexes with ferrozine, and the obtained data were presented in Table 7 (d). NRCG4 appeared to have a moderate ability to chelate ferrous ions in respect to ascorbic acid and BHT. NRCG4 inhibited the formation of Fe^2+^-ferrozine complex which means they are able to capture ferrous ion before ferrozine and prevented complex formation. NRCG4 recorded 35.43 ± 1.57 chelating percentage at minimum concentration and it was increased to 47.95 ± 1.96% with the highest one, in respect to vitamin C (62.99 ± 1.01% and 98.19 ± 1.82%, respectively) and BHT (52.41 ± 1.59% and 99.09 ± 0.90%, respectively) at the same concentrations (*P* < *0.05*). Values of IC_50_ for NRCG4, BHT, and vitamin C were as follows: 416.38, 18.87, and 16.36 μg/ ml, respectively.

### Lipid peroxidation Inhibition capacity

The capacity of NRCG4 to reduce lipid peroxidation was assessed using the thiocyanate technique. Linoleate radicals oxidized ferrous ion to hydroperoxides to generate ferric ion, which was then measured spectrophotometrically as a thiocyanate complex at 500 nm in the thiocyanate system. In the emulsion, NRCG4 revealed a preventive effect of linoleic acid against peroxidation (Table 7 (e)). NRCG4 inhibited linoleic acid peroxidation in a concentration-dependent manner; the lowest linoleic acid peroxidation inhibition activity (39.48 ± 0.52%) was reported with the lowest concentration (25 μg/ ml). In comparison to ascorbic acid, the greatest percentage)53.44 ± 1.78%) was provided with the maximum concentration (400 μg/ ml) (64.73 ± 2.20% and 100.00 ± 0.00 for the two concns., respectively) and BHT (60.31 ± 1.69% and 100.00 ± 0.00%, respectively). To prevent 50% of linoleic acid oxidation into peroxide, 190.45 μg/ml of NRCG4 was required whereas BHT at 19.65 μg/ml and ascorbic acid at 18.33 μg ml are required for the same effect.

### ***O***_***2***_*** radicals scavenging capacity***

NBT (yellow dye NBT^2+^) is reduced by superoxide anion (SOR) radicals formed in the PMS–NADH–NBT system by dissolving oxygen in the PMS–NADH coupling reaction. (Table 7 (f)) shows the proportion of generated SOR that was inhibited by NRCG4 at gradual concentrations when compared to reference materials at the same concentrations. NRCG4 exhibited SOR scavenging percentage ranged from 65.91 ± 2.09% at 25 μg/ml to 81.27 ± 2.73% at 400 μg/ml, with respect to those of ascorbic acid (66.21 ± 1.7% and 97.75 ± 2.25%, respectively) and BHT (73.22 ± 2.78% and 98.11 ± 1.89%, respectively). NRCG4 exhibited low IC_50_ value (14.67 μg/ ml) while BHT and ascorbic acid presented 11.01 μg/ml and 12.35 μg/ml, respectively, with significant differences among them (*P* < *0.05*).

### NO scavenging capacity

NO radical scavenging ability of NRCG4 was estimated by a SNP generating NO system. NO released from SNP in aqueous solution at physiological pH reacts with oxygen to generate nitrite ions which were measured. In the data presented in Table (7 (g)), NRCG4 occurred a significant decrease in nitrite liberated in SNP assay medium represented as moderate NO scavenging action compared to references materials. The NO scavenging capacity was concentration dependent. Therefore, the NO scavenging action of NRCG4 significantly rose from 17.89 ± 0.90% at 25 μg/ml to 58.64 ± 1.20% at the highest concentration (400 μg/ ml) which were lower than ascorbic acid (64.39 ± 1.61% and 93.27 ± 2.73% at the same concns, respectively) and BHT (65.99 ± 2.00% and 5.88 ± 3.56%, respectively, at the same two concentrations). The amount of NRCG4 to capture 50% of generated NO was 322.96 μg/ ml which was higher than those of both BHT (18.93 μg/ ml) and ascorbic acid (19.96 μg/ ml).

## Discussion

One of the primary factors contributing to an increase in neuronal disease (ND) instances around the world is the increase in modern man’s life expectancy, as well as the complexity of NDs and the lack of an acceptable NDs cure [46, 47]. Given the rise in the senior population, NDs will continue to be vital for scientists to effectively address [48]. In general, NDs cause cognitive and memory impairment, which impairs a person’s ability to breathe, move, and communicate. As a result, given the distress that NDs cause, effective treatments are required to improve patients’ quality of life [46].

The goal of this research was to find a new natural substance that may be used to treat Alzheimer’s disease to obviate the side effects of synthetic drugs. As a result of their unique living conditions, marine microorganisms frequently create bioactive compounds with novel activities and structures [49]. New medications rely on EPSs from marine microbes [9, 50, 51]. So, totally, ten isolates from the Red Sea, Hurghada, Egypt, were screened for the production of EPSs. Two isolates only could highly produce polysaccharide (NRCG1 and NRCG4). The most active isolate that has high acetyl cholinesterase inhibition activity (NRCG4) was subjected to morphological, physiological, and biochemical characteristics and by 16 s rRNA. Therefore, it was identified as *Streptomyces* sp. NRCG4 with accession number MK850242**.**

Our study demonstrated that NRCG4 could treat Alzheimer’s risk factors via inhibition of cholinesterase and tyrosinase as well as anti-inflammatory and antioxidant abilities. NRCG4 occurred a potential role in the suppression of Alzheimer’s disease biomarkers through its antioxidant (metal chelation, ROS scavenging, and radical activities) and anti-inflammatory characteristics of NRCG4 that assigned to its unique chemical composition.

Existing treatments for Alzheimer’s are expensive and come with several negative side effects [52]. Given that existing medicines can only treat the symptoms of NDs and that the disease is linked to aging, there is a pressing need to discover medications that can slow the progression of the disease [53].

L-DOPA, commonly known as Levodopa and L-3, 4-dihydroxyphenylalanine, is well known for its use as a treatment for vascular dementia. L-DOPA is the precursor for the neurotransmitter dopamine and is produced by the action of the enzyme tyrosinase on the amino acid tyrosine. In Alzheimer’s disease, aggregation of the peptide droves to the development of amyloid plaque formation followed by neurodegenerative changes. L-DOPA and dopamine can dissolve fibrils of a peptide and can inhibit the formation of protein tangles [54]. Unfortunately, tyrosinase can oxidize dopamine’s catechol ring to the extremely reactive dopamine-quinone. Dopamine transporter is inhibited by dopamine oxidation too, glutamate transport, and mitochondrial respiration. Thus, inhibition of tyrosinase can control and treat Alzheimer’s disease [55, 56]. One of the factors that contribute to the development of Alzheimer’s disease is the aggregation of Aβ protein. On the other hand the importance of non-steroidal anti-inflammatory medications (NSAIDs) as aggregation inhibitors has been demonstrated by numerous studies [57]. NSAIDs have even been shown in lab trials to reduce AD pathogenesis by inhibiting microglial activation or Aβ peptide deposition. [58–60].

Hasegawa et al. [61, 62] chose catecholaminergic neuronal cell lines that express tyrosinase in response to an external inducer such as doxycycline. In these cell lines, overexpression of the tyrosinase protein resulted in an increase in intracellular dopamine and reactive oxygen species (ROS), as well as the development of melanin pigments in neuronal somata, which leads to apoptotic cell death. Juneja et al. [63] used blastula protease-10 peptide as a tyrosinase-like mimic to study Type III-Copper proteins like tyrosinase’s intermediates and mechanisms. Furthermore, H_2_O_2_ activated the model and served as the second substrate in a bi-substrate reaction. Tyrosinase is a copper-containing enzyme that catalyzes the conversion of oxidized tyrosine to dopa and dopaquinone. Because of the copper structure in tyrosinase, various studies have looked into the potential function of metal-chelating substances such as kojic acid, catechol, gentisic acid, flavonol, and hydroxamic acid in tyrosinase activity management [64]. Generally, flavonoids with a hydroxyl group at A and B rings are very important tyrosinase inhibitors by Cu^2+^ chelation [65]. Polysaccharide NRCG4 represented chelating efficacy that was increased dependently by increasing concentration which could be one of its mechanisms to face Alzheimer progression or its treatment.

Many studies have focused on phenolic compounds in particular because of their antioxidant properties, which could be linked to neuroprotective properties by decreasing endogenous ROS generation, restoring mitochondrial membrane potential, enhancing superoxide dismutase activity, and elevating intracellular Ca^2+^ levels [66]. Taking this hypothesis into consideration, the antioxidant properties of examined polysaccharide NRCG4 could participate in the neuronal protection of cells from death and consequently save brain maintenance and treating Alzheimer’s signs.

A decrease in the neurotransmitter acetylcholine (Ach) is thought to be a key factor in the development of dementia in Alzheimer’s disease patients. As a result, people with Alzheimer’s disease must suppress both major forms of cholinesterase, AChE and BChE, in order to restore acetylcholine levels. In Alzheimer’s disease, inhibition of AChE plays a crucial role in increasing cholinergic transmission in the brain and lowering amyloid beta peptide (Aβ) aggregation and the generation of neurotoxic fibrils [35]. AChE protein consists of 531 amino acid residues with an ellipsoid shape. AChE has two binding sites; the first is involved in the interaction of positively charged function group as nitrogen in alkaloid derivatives, and the second can interact with other non-alkaloid components as phenols [67]. The considered NRCG4 in this study was characterized to have 16% uronic acid and the mannuronic acid was constituted at 1.2 molar ratio which may accept the negative charge for molecule that may interact with the other side of acetylcholinesterase causing enzyme block showed in enzyme inhibitory action.

Materials that have antioxidant activity and acetylcholinesterase inhibitory action are considered promising in treating Alzheimer’s disease. For example, *Centella asiatica* (L.) Urb. is a valuable medicinal plant used to cure cognitive ailments and mental health issues, according to studies. A recent study found that an ethanolic extract of *C. asiatica* significantly reduces oxidative stress and AChE activity in the brain produced by D-gal, while also preserving normal cellular architecture in the hippocampus and cortical areas. The study’s authors found that *C. asiatica* can protect the brain from D-negative gal’s consequences, such as memory loss [68]. In our presented study, NRCG4 contains many sugars like galactose, L-arabinose which may be the cause of higher selectivity to AChE than BuChE.

*Streptomyces* sp. is considered common host bacteria for industrial and medicinal applications. Furthermore, some *Streptomyces* produce EPSs with biological functions [51, 69, 70]. The majority of marine bacteria created heteropolysaccharides, which are polysaccharides made up of different monosaccharides. Additionally, it has been discovered that glucose and mannose have receptors on macrophages that are unique for tumor immunology [71]. The results of molecular weight are similar to those of Sutherland [72], who noted that a major fraction of EPSs given from marine sources have an average molecular weight of 1–3 × 10^5^ Da. The molecular weight and biological activity of EPSs are related; the activity is dependent on the number of molecules as well as the degree of branching and conformation [73].

It is believed that Alzheimer risk factors including environmental, biological, and genetic factors associated with elevating level of inflammatory biomarkers that can promote advanced cognitive deterioration. In addition, it was demonstrated that the development of different central nervous system (CNS) diseases, e.g., Alzheimer’s disease, is related to neuroinflammation. Inflammatory process development implicates a widely spread range of molecular interactions which represent important CNS changes outcomes. These changes contribute to CNS regulation and function impairment that increased the level of inflammatory marker [74]. When the inflammatory cascade was initiated the neuroinflammatory process becomes highly activated to make further cellular damage to loss their function which is accompanied with neurofibrillary tangles and amyloid plaques formation, Alzheimer’s hallmarks [75]. Therefore, the anti-inflammatory effect of NRCG4 was determined. The investigated polysaccharide exerted selective inhibitory action against COX-2 which led to decreasing the production of inflammatory cytokines in cells that is recommended as targeted mechanism in treating Alzheimer’s disease and this selectivity could keep brain and body maintenance.

## Conclusion

In the current study, the exopolysaccharide was produced from *Streptomyces* sp. NRCG4 which isolated from marine sample of the Red Sea, Hurghada, Egypt. NRCG4 exhibited promising antioxidant characteristics (metal chelation, ROS scavenging, radical scavenging capability, and inhibition of lipid peroxide production) and selective inhibitory effects against COX-2, referring that it could be a promising natural raw material to treat inflammation-related diseases as Alzheimer’s. Additionally, it possessed tyrosinase inhibitory action and neurotransmitters degradation inhibitory action that supports its role as pronounced Alzheimer’s treating agent. The promising therapeutic features of NRCG4 may attribute to its structure and features.

## Data Availability

All data generated or analyzed during this study are included in this article.

## Abbreviations

EPS: Exopolysaccharides
°C: Degree Celsius
mg: Milligram
µg: Microgram
ml: Milliliter
FTIR: Fourier-transform infrared
HPGPC: High-performance gel permeation chromatography
HPLC: High-performance liquid chromatography
DPPH: 1,1-Diphenyl-2-picryl-hydrazyl
BHT: Butylated hydroxytoluene
ABTS: 2, 2-Azino-bis (3-ethylbenz-thiazoline-6-sulfonic acid)
NADH: Nicotinamide adenine dinucleotide
NBT: Nitroblue tetrazolium
PMS: Phenazine methosulphate
SNP: Sodium nitroprusside
TCA: Trichloroacetic acid
ROS: Reactive Oxygen Species
DTNB: 5,5-Dithiobis(2-nitrobenzoic acid)
